# Highly Efficient CYP167A1 (EpoK) dependent Epothilone B Formation and Production of 7-Ketone Epothilone D as a New Epothilone Derivative

**DOI:** 10.1038/srep14881

**Published:** 2015-10-08

**Authors:** Fredy Kern, Tobias K. F. Dier, Yogan Khatri, Kerstin M. Ewen, Jean-Pierre Jacquot, Dietrich A. Volmer, Rita Bernhardt

**Affiliations:** 1 Department of Biochemistry, Saarland University, 66123 Saarbrücken, Germany; 2 Institute of Bioanalytical Chemistry, Saarland University, 66123 Saarbrücken, Germany; 3 Unité Mixte de Recherches, 1136 Interaction arbres microorganismes INRA, Nancy University, 54506 Vandoeuvre-lès-Nancy cedex, France

## Abstract

Since their discovery in the soil bacterium *Sorangium cellulosum*, epothilones have emerged as a valuable substance class with promising anti-tumor activity. Because of their benefits in the treatment of cancer and neurodegenerative diseases, epothilones are targets for drug design and pharmaceutical research. The final step of their biosynthesis – a cytochrome P450 mediated epoxidation of epothilone C/D to A/B by CYP167A1 (EpoK) – needs significant improvement, in particular regarding the efficiency of its redox partners. Therefore, we have investigated the ability of various hetero- and homologous redox partners to transfer electrons to EpoK. Hereby, a new hybrid system was established with conversion rates eleven times higher and V_max_ of more than seven orders of magnitudes higher as compared with the previously described spinach redox chain. This hybrid system is the most efficient redox chain for EpoK described to date. Furthermore, P450s from So ce56 were identified which are able to convert epothilone D to 14-OH, 21-OH, 26-OH epothilone D and 7-ketone epothilone D. The latter one represents a novel epothilone derivative and is a suitable candidate for pharmacological tests. The results revealed myxobacterial P450s from *S. cellulosum* So ce56 as promising candidates for protein engineering for biotechnological production of epothilone derivatives.

In 2012, the World Human Organization’s global target of reducing premature mortality from non-communicable diseases (e.g. cancer) by 25% was set to be achieved by 2025^1^. To reach this ambitious target, population-based cancer registries and surveillance systems^2^ as well as fundamental cancer research are essential. For decades, chemotherapy has been the leading therapeutic approach in the treatment of cancer. Interestingly, more than 60% of anticancer agents currently in use are derived from natural sources, including plants, marine organisms and microorganisms^3^. Among these compounds, agents blocking mitosis rate by targeting microtubules belong to the most efficient anti-cancer drugs identified to date^4^. One member of the group of microtubule-stabilizing agents are epothilones, which were first discovered in 1987 by Gerth and coworkers as antifungal compounds naturally produced by the soil bacterium *Sorangium cellulosum*^5^,^6^. After the discovery of the cytotoxic activity of epothilones against a number of tumor cell lines in 1995^7^, many studies and clinical trials with epothilones were published, with epothilone D and B emerging as the most promising candidates for treatment of cancer. With respect to favorable characteristics as anticancer drugs, only derivatives of epothilone D and B have reached the stage of clinical investigation. Among those, Ixabepilone (an epothilone B analogue) is the only approved drug in cancer therapy to date^8^. Moreover, further studies have recently revealed additional benefits for treatment of Alzheimer’s and Parkinson’s disease with epothilone D^9^,^10^. Because of the variety of possible medical applications, epothilone D and its derivatives are most interesting targets for pharmaceutical and medical investigations.

The biosynthesis of epothilone D was first proposed by sequence determination of the biosynthetic gene cluster in *S. cellulosum* So ce90^11^ and evaluated with the cloning of the complete gene cluster from *S. cellulosum* SMP44 into *S. coelicolor*^12^. It was demonstrated that the myxobacterial CYP167A1 (EpoK) is responsible for the final step of epothilone biosynthesis (epoxidation of epothilone D to B (Fig. 1), and epothilone C to A, respectively). In general, cytochrome P450 enzymes (P450) are versatile enzymes catalyzing a variety of reactions^13^, a characteristic that makes them essential components of biotechnological and pharmaceutical research^14^. But P450s belong to the external monooxygenases and thus require electron supply from external redox partners^15^. Because neither the natural nor an efficient heterologous redox system supporting EpoK are known, the establishment of a whole-cell system in *E. coli* could not be achieved to date.

Although it was reported early on that an artificial redox chain consisting of spinach ferredoxin and ferredoxin reductase can support EpoK activity, the efficiency of the reaction was rather low. For the C-terminal his-tagged EpoK (1.5 *μ*M), only 60% yields were observed after one hour at 30 °C when 100 *μ*M epothilone D was used as substrate^12^. Putidaredoxin and putidaredoxin reductase from *Pseudomonas putida* also turned out not to be efficient enough to be a good alternative for the spinach system^16^. Therefore, it is paramount to establish an efficient redox chain to unlock the biotechnological potential of EpoK.

In this study, we first investigated homo- and heterologous electron transfer systems for an *in vitro* conversion of epothilone D by EpoK. Therefore, we studied *bovine* adrenodoxin (Adx_4-108_) and adrenodoxin reductase (AdR), electron-transfer-protein 1 (Etp1^fd^) and its autologous adrenodoxin reductase homologue 1 (Arh1) from *Schizosaccharomyces pombe*, and myxobacterial ferredoxins and ferredoxin reductase from *S. cellulosum* So ce56 (Fdx2/FdR_B and Fdx8/FdR_B), as well as a novel hybrid electron transfer system for P450s, ferredoxin (SynFdx) from *Synechocystis* and ferredoxin NADP^+^ reductase (FNR) from *Chlamydomonas reinhardtii*.

In addition, we examined selected P450s from the related strain *S. cellulosum* So ce56 for a conversion of epothilone D. P450s of this strain were recently investigated by our group and exhibited novel functionalities and a broad substrate range^17^,^18^,^19^,^20^. Bioinformatics study revealed some of these P450s to be closely related to EpoK. As a result, the members of the CYP109, CYP260, CYP264 and CYP267 families as well as CYP265A1 and CYP266A1 from So ce56 were selected and implemented for *in vitro* conversions. The resulting products were subsequently analyzed via HPLC and LC-MS/MS. All structure proposals were tentatively assigned by LC-MS/MS and proposed collision-induced dissociation spectra are presented.

## Results

### Investigated electron transfer proteins: important characteristics

During our studies, several homologous and heterologous electron transfer systems were investigated. The general characteristics of the respective components are listed in Table 1 for ferredoxins and Table 2 for reductases, respectively. It is noteworthy that the redox potential of ferredoxins is decreasing from −344 mV for Adx_4-108_ to −353 mV for Etp1^fd^ and to a redox potential of −380 mV for ferredoxin (SynFdx) from *Synechocystis*. The ferredoxins Adx_4-108_, Etp1^fd^ and SynFdx belong to the [2Fe-2S]-type and Fdx2 and Fdx8 from So ce56 to the [3Fe-4S]-type ferredoxins. Moreover, both eukaryotic reductases, AdR and Arh1, have a molecular weight of around 50 kDa, almost twice the values for the comparatively small reductase FdR_B from *S. cellulosum* So ce56.

### *In vitro* conversions of epothilone D by EpoK

**Figure 2:**
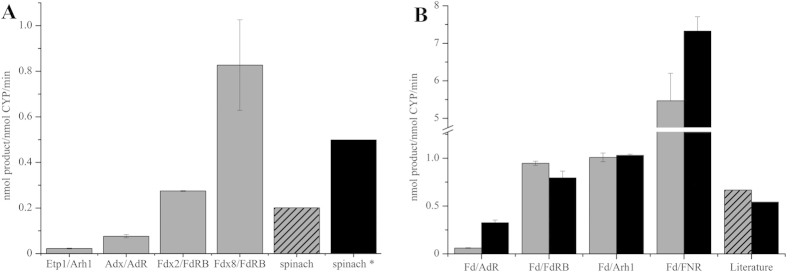
(**A**) Epothilone D conversions by EpoK with different electron transfer partners (spinach: ratio 1:100:0.025U; spinach*: ratio 1:100:0.125U for P450:Fdx:FdR; reductase in units); (**B**) Epothilone D conversions with EpoK, ferredoxin SynFdx (Fd) from *Synechocystis* and selected reductases (ratio 1:10:1 in grey bars and ratio 1:20:3 in black bars); bars designated as “Literature” were calculated from published data using spinach redox system: dashed^12^ and black bar^21^

EpoK was tested with a variety of electron transfer partners as shown in Fig. 2A. The redox systems Etp1^fd^/Arh1 from *S. pombe* and Adx_4-108_/AdR from *Bos taurus* showed conversion rates below 0.1 nmol product per nmol P450 per min. The ferredoxins Fdx2 and Fdx8 with their autologous reductase FdR_B showed conversion rates of 0.3 and 0.8 nmol product per nmol P450 per min, respectively. Investigations with spinach redox partners analogous to Tang *et al*.^12^ lead to conversion rates of 0.2 nmol product per nmol P450 per min for the described ratio (1.5:100:0.025U for P450:Fdx:FdR). Using optimized reaction conditions (five times higher reductase amount), 0.5 nmol product per nmol P450 per min were observed. Summarizing the results, the utilization of the homologous redox partners Fdx2/FdR_B and Fdx8/FdR_B resulted in an up to 4 times higher conversion rate compared to our results with the spinach redox system, thus presenting a more efficient redox chain for EpoK compared to published results.

**Figure 3:**
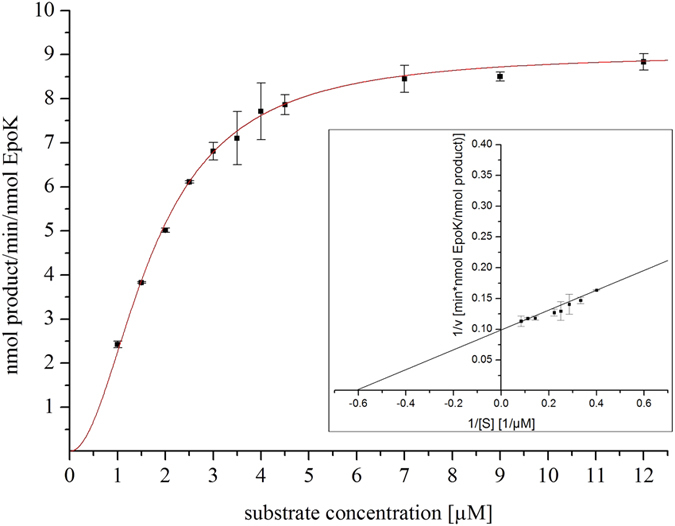
Kinetic studies on epothilone B formation supported by EpoK/SynFdx/FNR hybrid system (ratio 1:20:3 for EpoK:SynFdx:FNR). The Hill model with n_H_ = 2 resulted in a sigmoidal fit for EpoK kinetics with a coefficient of determination (R^2^) of 0.99. The inset shows the Lineweaver-Burk plot of the data with R^2^ = 0.99.

To evaluate the suitability of SynFdx as electron mediator for EpoK, different combinations and ratios of SynFdx with selected reductases were investigated. SynFdx was combined with different heterologous reductases in two different ratios (1:10:1 and 1:20:3 for P450:SynFdx:reductase). To compare the efficiency of the spinach redox system with our results, we calculated conversion rates [nmol product per min per nmol P450] from published data of epothilone D conversion by EpoK^12^,^21^ to include them into Fig. 2B. All tested combinations, except SynFdx with *bovine* AdR, resulted in higher conversion rates than described in previous publications. When FdR_B from *S. cellulosum* So ce56 and Arh1 from *S. pombe* were used as electron donor for SynFdx, slightly higher rates compared to the spinach system were observed. However, the hybrid redox system containing ferredoxin from *Synechocystis* and FNR from *C. reinhardtii* yielded eight to eleven times (depending on component ratio) higher conversion rates *in vitro*, compared with the values calculated for spinach Fdx/FNR. Additionally, kinetic studies of the epoxidation of epothilone D were performed using the ratio 1:20:3 for the EpoK/SynFdx/FNR system. A v_max_ value of 9.03 ± 0.17 nmol product min^−1^ nmol^−1^ EpoK and K_1/2_ = 1.73 ± 0.05 *μ*M were obtained by using the Hill-equation (Fig. 3). The replotted Lineweaver-Burk plot of the data (Fig. 3 inset) also showed the similar values of v_max_ and K_m_ of 10.10 nmol product min^−1^ nmol^−1^ EpoK and 1.63 *μ*M, respectively. Compared with literature data (v_max_ = 5.6 · 10^−7^ nmol product min^−1^ nmol^−1^ EpoK and K_m_ = 1.6 *μ*M^16^), the v_max_ value was increased by seven orders of magnitude (10^7^) with a similar K_m_ value. Thus, a highly efficient redox chain was established for EpoK.

### Bioinformatics identification of potent epothilone D monooxygenases in *S. cellulosum* So ce56

**Figure 4:**
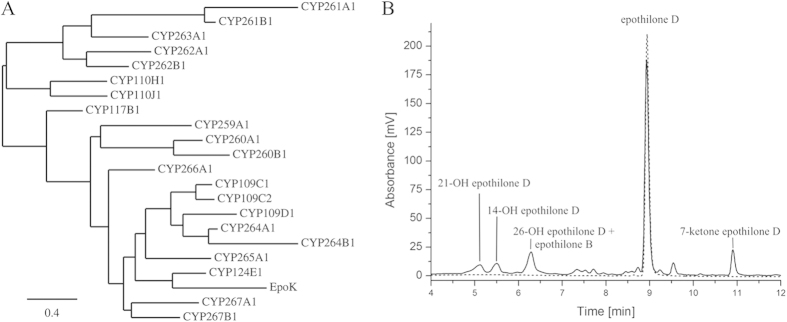
(**A**) Phylogenetic tree of EpoK and CYPome of So ce56. The tree was constructed by using protein sequences and the “One Click” mode of the online phylogenetic analysis tool from Information Génomique et Structurale, Marseille, France. The bar in the left corner indicates 0.4 amino acid substitutions per amino acid for the branch length^65^. (**B**) HPLC chromatogram of epothilone D (dashed line) and its conversion by CYP267B1/Fdx8/FdR_B (solid line).

To find additional P450s able to convert epothilones, we investigated the CYPome of So ce56. The phylogenetic comparison of EpoK with the CYPome of So ce56 revealed several P450s of So ce56 closely related to EpoK from So ce90 (Fig. 4A). Among the CYPome of So ce56, the greatest protein sequence homology (approximately 50%) and identity (≥30%) to EpoK was found for CYP124E1, CYP266A1, CYP267A1 and CYP267B1.

### *In vitro* conversions of epothilone D with selected P450s from *S. cellulosum* So ce56

CYP266A1, CYP267A1 and CYP267B1 of *S. cellulosum* So ce56 were tested with respect to their ability to convert epothilone D. Due to low expression levels^22^, CYP124E1 was not studied. But, in addition to the P450 members with the greatest homologies to EpoK, also CYP109, CYP260 and CYP264 families as well as CYP265A1 were further investigated regarding a potential conversion of epothilone D. For this, reconstituted *in vitro* systems analogous to the ones used for EpoK-dependent epothilone D conversion were used. Interestingly, in contrast to EpoK from So ce90, efforts to apply the hybrid system SynFdx/FNR as an electron transfer system for P450s from So ce56 were unsuccessful. As a result, *bovine* Adx_4-108_/AdR as well as the autologous Fdx2/FdR_B and Fdx8/FdR_B redox chains were used as described previously^22^,^23^.

As shown in Table 3, CYP265A1 and CYP266A1 are able to convert epothilone D, with this compound representing the first presently identified substrate for each of these P450s. Interestingly, from the CYP267 family, only CYP267B1 showed activity towards epothilone D. Thus, all of the tested P450s closely related to EpoK, with the exception of CYP267A1, are able to convert epothilone D. In contrast, for the respective CYP109, CYP260 and CYP264 family members, no conversion of epothilone D was observed neither with the heterologous redox partners Adx_4-108_/AdR nor with the autologous electron transfer proteins Fdx2/FdR_B and Fdx8/FdR_B.

Both, CYP265A1 and CYP266A1 were able to catalyze a hydroxylation of epothilone D in position 14 (Table 3, details on identification and characterization of the products via LC-MS/MS see below). Three of the tested redox systems (Adx_4-108_/AdR, Fdx2/FdR_B and Fdx8/FdR_B) were able to transfer electrons to CYP265A1, with CYP265A1/Fdx8/FdR_B showing the highest conversion yield (6% 14-OH epothilone D). CYP266A1 converts epothilone D to 14-OH epothilone D most efficiently when supported by Fdx2/FdR_B (6.9% conversion), whereas *bovine* Adx_4-108_/AdR was not able to transfer electrons to CYP266A1. The system yielding the highest total conversion of epothilone D was found to be the autologous CYP267B1/Fdx8/FdR_B system. Most remarkably, the product pattern of CYP267B1 revealed five products of epothilone D conversion (Fig. 4B). Besides 14-OH epothilone D (4.8% conversion) and 21-OH epothilone D (5.5% conversion), 26-OH epothilone D and epothilone B were observed and gave a combined conversion of 11.4%. Due to their chemical similarity, 26-OH epothilone and epothilone B display the same retention time of 6.3 min (Table 3 and Fig. 4B). However, only small amounts of epothilone B were formed (supplemental Figure S2). Among the apolar products, only the product at 10.9 min has been characterized and revealed 7-ketone epothilone D (8.7% conversion). These products have not been characterized for P450-derived catalysis to date and thus represent novel products. The chemical structures of the identified products are shown in supplemental Figure S1.

### LC-MS/MS identification of products formed during *in vitro* conversion of epothilone D by CYP265A1, CYP266A1 and CYP267B1

**Figure 5:**
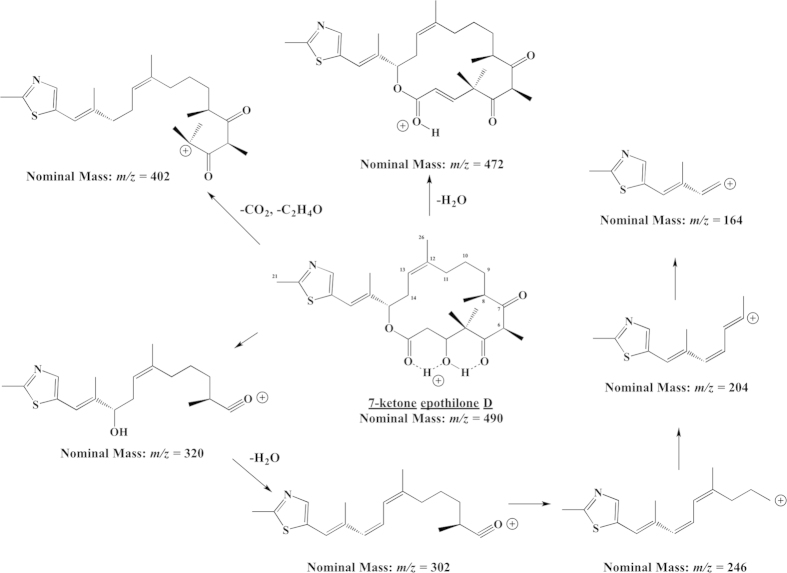
Proposed collision-induced dissociation spectrum of 7-ketone epothilone D (*m/z* 490). Loss of water at position C-3 results in a fragment with *m/z* 472. The fragment ion at *m/z* 402 appears after the loss of CO_2_ and C_2_H_4_O. Step-wise fragmentation of the precursor ion is proposed and presented counterclockwise.

Product identification for the *in vitro* conversion of epothilone D by CYP265A1, CYP266A1 and CYP267B1 was performed by LC-MS/MS analysis. As shown in supplemental Table S1, five epothilone derivatives were obtained and characterized during this study. To elucidate the structures of conversion products and to identify sites of hydroxylation or oxidation, CID of the analogous epothilone B compound was performed for comparison purposes. Blum *et al*. have shown that single or multiple losses of small molecules such as water (18 Da) dominated the CID spectra of epothilone B^24^. Losses of water originated at positions C-2 and C-3 and/or C-6 and C-7 from the precursor ion or as final step in serial dissociations. Further cleavages occurred at the carbon-oxygen bond at position 15 with formal loss of CO_2_ (44 Da). Subsequent C-C cleavages at various positions in addition to C-O dissociation led to a number of characteristic ions (supplemental Table S1) in our experiments. Several of these product ions (marked with *) were also present in the CID spectra of the other products of epothilone D conversion. Tentative structure assignments of the conversion products A to D to the general epothilone substance class were therefore readily possible. Substance-specific ions such as *m/z* 168, 206 and 220 permitted identification of hydroxylation reactions. In addition, the presence of these ions as well as the precursor ion at *m/z* 508 pointed to a single hydroxylation site located directly at or near the thiazole ring. The proposed structures of the product ions listed in the supplemental Table S1 allowed us to tentatively assign the products A and B as 21-OH epothilone D, product C as 14-OH epothilone D, and product D as 26-OH epothilone D (supplemental Figure S2). Conversion product E corresponded to an oxidation product of epothilone B with a specific fragmentation scheme (supplemental Figure S3), which was assigned to the general epothilone substance class. Product E was identified as 7-ketone epothilone D and the proposed collision-induced scheme is illustrated in Fig. 5.

## Discussion

Epothilones belong to a family of novel microtubule-stabilizing agents, which inhibit mitosis. Therefore they are interesting compounds for cancer research and treatment^25^. Their benefits over e.g. paclitaxel as anti-tumor agents are numerous. For example, some studies revealed higher water solubility^6^ and inhibition of cancer cells resistant to various other chemotherapeutic agents^7^. During further studies, additional benefits of a treatment with epothilone D were described. Low-dose epothilone D treatment of aged PS19 mice with tau protein malformation in brain neurons (characteristic for Alzheimer disease) led to promising reduction of axonal dysfunction and neurotoxicity^10^. For Parkinson’s disease, studies with epothilone D showed a rescue of chemically induced microtubule defects^9^.

CYP167A1 (EpoK) is a P450 enzyme located downstream of the polyketide synthase (PKS) system of *S. cellulosum* So ce90. It is responsible for the last step in epothilone biosynthesis in this organism, resulting in epoxidation of epothilone D to epothilone B^21^. However, detailed information on the redox partners of EpoK, necessary for epothilone A/B formation in *S. cellulosum* So ce90, are not available. Generally, the P450 systems are classified (class I-X) according to the number, structure and topology of the protein components involved in the electron transfer to the P450 enzyme^15^. Most bacterial P450 systems belong to class I, in which the systems are composed of three separate proteins. Currently, the only electron transfer system described to support EpoK consists of spinach ferredoxin and spinach ferredoxin NADP^+^ reductase. In 2000, Tang *et al*. observed a 60% conversion of epothilone D to B within one hour, using the redox chain from spinach. Attempts to replicate these results during our study resulted in considerably lower yields and conversions rates than described^12^. To find new efficient redox partners for EpoK, different homo- and heterologous class I electron transfer proteins were investigated in the present study.

Several investigations by our group have revealed the *bovine* Adx_4-108_/AdR electron system as high-profile partner for efficient P450 *in vitro* and whole-cell experiments with a broad spectrum of P450s of prokaryotic origins supported^23^,^26^,^27^. Here, we found that *bovine* Adx_4-108_/AdR is also able to transfer electrons to the heterologous EpoK from *S. cellulosum* So ce90. However, conversion of epothilone D by the EpoK/Adx_4-108_/AdR system led to conversion rates significantly below the described and replicated results with the spinach system. For the electron transfer proteins Etp1^fd^ and Arh1 from *S. pombe*, yields and conversion rates for the investigated EpoK/Etp1^fd^/Arh1 system were even lower. Despite their ability to provide mammalian P450s with reduction equivalents^28^,^29^, the application of Etp1^fd^/Arh1 (or Adx_4-108_/AdR) as electron donor system for EpoK thus remains limited.

After Schneiker *et al*. sequenced the genome of *S. cellulosum* So ce56 in 2007^30^, our group characterized not only 21 P450s^22^ but also 8 ferredoxins and 2 ferredoxin reductases^31^. It was revealed that the combination of Fdx2 and Fdx8 with FdR_B offers suitable electron transfer activities to many myxobacterial P450s, making the So ce56 redox pairs Fdx2/FdR_B and Fdx8/FdR_B interesting targets for *in vitro* studies with EpoK from So ce90. During this study, it was shown that the utilization of myxobacterial ferredoxin and ferredoxin reductase leads to higher yields and conversion rates compared with bovine Adx_4-108_/AdR or Etp1^fd^/Arh1 from *S. pombe*. With regard to the yields described by Tang *et al*. and Julien *et al*.^12^,^21^ and conversion rates calculated based on these data, the homologous EpoK/Fdx8/FdR_B system revealed an efficiency to convert epothilone D comparable with the results published for the spinach redox chain. Bioinformatics study on the genes of Fdx2, Fdx8 and FdR_B from So ce56 revealed high identity (93.1%, 78.2% and 92.7%) to putative ferredoxin and ferredoxin reductase genes of *S. cellulosum* So0157-2 (supplemental Table S2). Sequenced in 2013 by Han *et al*.^32^, the So0157-2 strain of *S. cellulosum* is an alkaline-adaptive producer of glycosylated epothilone A and B derivatives with high relationship to the not yet sequenced So ce90 strain^33^. Thus, the detection of ferredoxin and ferredoxin reductase genes, which are highly similar to the respective genes from So ce56, whose gene products were shown to support EpoK activity, is the first step to identify and ultimately purify the natural redox partners of EpoK from *S. cellulosum* So ce90.

In order to further optimize *in vitro* conversions catalyzed by EpoK, novel electron transfer proteins were also tested. We selected ferredoxin (SynFdx) from the unicellular cyanobacterium *Synechocystis* sp PCC6803 as electron mediator. *Synechocystis* is a photoautotrophic organism capable of oxygen-producing photosynthesis^34^ and is able to synthesize two different Photosystem-I electron acceptors, a [2Fe-2S] ferredoxin and flavodoxin, both first purified and characterized by Bottin *et al*. in 1992^35^. There are eight other ferredoxins in *Synechocystis*, but the ferredoxin (SynFdx) chosen for this study presently received the most attention as a result of its diversified involvement in redox processes, such as cyclic photophosphorylation, nitrogen assimilation, biosynthesis of glutamate and chlorophyll, sulfite reduction and fatty acid metabolism^36^. As an additional novel member for an *in vitro* P450 electron transport chain, ferredoxin NADP^+^ reductase (FNR) from the unicellular green alga *C. reinhardtii* was included in our studies. The selected FNR, a protein naturally involved in the NADP^+^ reduction in the chloroplast stroma^37^, was first isolated and characterized by Decottignies *et al*. in 1995^38^. A comparison of plant and bacterial ferredoxin-NAD(P)^+^ reductases led to the conclusion that plant ferredoxin-NAD(P)^+^ reductases evolved higher efficiency and turnover rates to operate in rapid metabolic pathways such as oxygenic photosynthesis^39^, recommending FNR from *C. reinhardtii* as an interesting partner for *in vitro* investigations.

The SynFdx/FNR/NADPH hybrid system was recently tested for cytochrome c reduction^40^. However, the combination of the SynFdx/FNR system with P450s in *in vitro* experiments remained unexplored. Therefore, we employed two different ratios of the P450 and redox partners (1:10:1 or 1:20:3 for EpoK:SynFdx:FNR) and found that the latter ratio of the hybrid system revealed a significant increase of conversion rates and a complete conversion of 100 *μ*M epothilone D to B within one hour. A change in the ratio of EpoK:SynFdx:FNR did not alter the regioselectivity of epothilone D epoxidation. For an efficient electron transfer chain, the formation of short-lived complexes is essential, dictated and controlled by the details of non-covalent interactions^41^. Optimal redox partners can be advantageous to increase the activities of recombinant P450 systems^14^. Therefore, it is noteworthy that although a reconstituted system with components originating from very different organisms (a myxobacterial P450, a cyanobacterial ferredoxin from *Synechocystis* and a plant ferredoxin NADP^+^ reductase from *C. reinhardtii*) was studied, a high efficiency for *in vitro* conversions was detected.

As shown in Fig. 3, kinetic studies of the EpoK/SynFdx/FNR system resulted in sigmoidal kinetics. It can be assumed that allosteric effects are responsible for the Hill coefficient n_H_ = 2. Two substrate molecules in the active site, as known for CYP3A4 or CYP2C9^42^ and already proposed for EpoK^16^, and the special feature of EpoK to be renaturated by its natural substrate epothilone D^16^, support this assumption. Our regenerated Lineweaver-Burk plot showed that the observed K_m_ value is similar to the one reported in the literature^16^. However, the observed v_max_ value is remarkably more than seven orders of magnitude (10^7^) higher than the one described for EpoK supported by the spinach redox partners^16^, thus rendering the EpoK/SynFdx/FNR system much more attractive for biotechnological application. With possible further improvements of the system in mind, it is also interesting to consider the characteristics of the tested electron transfer partners and their implications for the system: spinach ferredoxin, the first and - until this study - only ferredoxin reportedly supporting EpoK, has a low redox potential of −415 mV^43^. Interestingly, SynFdx, which exhibited the best conversion rates in this study (Fig. 2B), shows the most negative redox potential compared with Adx_4-108_ and Etp1^fd^ (Table 1). It is assumed that EpoK generally requires an electron mediator with a rather low redox potential. However, the redox potential is clearly not the only determinant for efficient electron transfer to EpoK since spinach ferredoxin is characterized by a yet lower redox potential than SynFdx.

Moreover, with SynFdx from the phototrophic *Synechocystis* acting as electron mediator for EpoK, a new way of epothilone B synthesis is imaginable. Recently, Jensen and co-workers described a light-driven hydroxylation of hydrocarbons using mycobacterial CYP124 in combination with spinach Fdx and photosystem I from *Hordeum vulgare*^44^. Applying SynFdx and photosystem I from *Synechocystis* as a redox chain for EpoK, a new interesting possibility of a light-driven, NADPH-independent epoxidation of epothilone D might be accessible.

Besides investigation of redox partners for EpoK and conversion of epothilone D to B, we investigated further opportunities for derivatization of epothilone D during this study. As mentioned before, the broad range of applications highlights epothilones as interesting targets for drug design, cancer therapy and pharmaceutical research. The availability and production of epothilones is therefore of great interest to the pharmaceutical industry. In addition to the recently improved extraction methods for epothilones from *S. cellulosum* fermentation broth^45^, total chemical synthesis of epothilones^46^, precursor-directed biosynthesis^47^ and heterologous production of epothilones in microorganisms^48^,^49^ are fields of research. Furthermore, also novel derivatives of epothilones are desirable compounds^8^. Investigations towards epothilone derivatives were described using chemical modifications^50^, extraction of derivatives from different *S. cellulosum* strains^51^, biotransformations with *Amycolata autotrophica*^52^ or selective conversion of epothilone B to epothilone F with a P450 hydroxylase from *Amycolatopsis orientalis*^53^. During the past few years, P450s of *S. cellulosum* So ce56 have revealed a great potential for industrial and biotechnological applications^17^,^20^,^22^,^23^,^54^. In order to produce potentially useful epothilone derivatives, we selected the members of CYP109, CYP260, CYP264 and CYP267 families as well as CYP265A1 and CYP266A1 from *S. cellulosum* So ce56 to test their ability to convert epothilone D. For the study described here, three different redox systems (P450/Adx_4-108_/AdR, P450/Fdx2/FdR_B and P450/Fdx8/FdR_B) were tested with the P450s mentioned above.

For the members of CYP109, CYP260 and CYP264 families and for CYP267A1, no conversions of epothilone D with any of the redox systems were observed. In contrast, CYP265A1, CYP266A1 and CYP267B1, which were showing high similarity to EpoK (Fig. 4A), were found to be able to convert epothilone D, in which CYP265A1 showed almost similar preference for Fdx2/FdR_B and Fdx8/FdR_B followed by Adx_4-108_/AdR (Table 3). However, CYP266A1 preferred Fdx2/FdR_B followed by Fdx8/FdR_B and has no activity with Adx_4-108_/AdR (Table 3). Although the conversion percentage was different, there was no change in the regioselectivity of hydroxylation, thus highlighting the use of the autologous redox systems for P450s of *S. cellulosum* So ce56 in future whole-cell studies. It is, however, interesting to mention that the use of different redox partners for CYP267B1 revealed different regioselectivity of epothilone D oxidation, in which the combination of CYP267B1 with Fdx8/FdR_B and Adx_4-108_/AdR preferred the 26-OH epothilone D/epothilone B product followed by 7-ketone epothilone D, whereas Fdx2/FdR_B showed a preference for 7-ketone epothilone D formation (Table 3). It can be assumed that the different ferredoxins mediate the electron flow to CYP267B1 with different efficiency and are, therefore, affecting the product distribution. An effect of different redox partner combinations on the regioselectivity of substrate hydroxylation has also been observed for P450 MycG^55^.

The products of epothilone D conversion, 14-OH, 21-OH and 26-OH epothilone D, can currently only be obtained via biotransformation of epothilone D by *Amycolata autotrophica*^52^, as natural products of So ce90/B2 and So ce90/D13^56^ or via chemical synthesis^57^. Cytotoxic activity data (IC50, MCF7 breast cancer cell line) of 14-OH (29 nM), 21-OH (23 nM) and 26-OH epothilone D (95 nM) were published earlier, with values higher than for epothilone D (9 nM) or B (0.5 nM), respectively^52^. However, for the first time, we found P450s, which open the possibility of a P450-derived production of 14-OH, 21-OH and 26-OH epothilone D to perform further studies with those derivatives. Most interestingly, CYP267B1 was able to oxidize epothilone D at position C-7 to 7-ketone epothilone D (Table 3). The positions in the epothilone molecule surrounding position C-7 have been shown to be of importance for the pharmacological effect of this compound. Thus, the removal/insertion of the methyl group, the reduction of the C = O group or the extension/reduction of the size of the epothilone D ring result in a less cytotoxic effect^58^. In contrast, the function of substitutions at position C-7 have currently not been analyzed and need to be further investigated to be able to find a potential pharmacologically active derivative of epothilones.

In summary, our results demonstrate that the redox pair Fdx8/FdR_B from *S. cellulosum* So ce56 efficiently supports the epothilone D conversion catalyzed by EpoK thus indicating a potential similarity to natural redox partners from So ce90. This hypothesis is supported both by experimental results and bioinformatics study. Apart from Fdx8/FdR_B, several other efficient redox partners were also identified in this study, which enable for the first time the implementation of EpoK in semisynthetic^47^, biotechnological^49^ or putatively even light-dependent^44^ epothilone B production. Following the hybrid system of *Anabaena* ferredoxin-NADP^+^ reductase and *bovine* Adx described in 2003^59^, the newly established *in vitro* hybrid system consisting of SynFdx from *Synechocystis* and FNR from *C. reinhardtii* reveals great potential for future EpoK *in vitro* and whole-cell studies and brings hybrid systems for P450 applications back into discussion.

With regard to the derivatization of epothilone D, eleven myxobacterial P450s have been investigated concerning their respective potential of converting epothilones and three of them were identified to convert epothilone D. Thereby, 14-OH, 21-OH and 26-OH epothilone D were found as novel P450-derived products. Additionally, a new epothilone derivative (7-ketone epothilone D) with a potential anti-tumor activity available by CYP267B1-dependent conversion was obtained (Fig. 5). Along with the autologous redox partners from *S. cellulosum* So ce56, further studies on protein engineering targeting higher yields and selectivity could lead to an important biotechnological application of myxobacterial P450s.

## Methods

### Chemicals

Phusion^TM^ High Fidelity DNA polymerase was purchased from Finnzymes (Espoo, Finland), Fast-Link^TM^ and DNA ligation kit from EPICENTRE Biotechnologies (Madison, WI, USA). Restriction enzymes were obtained from Promega (Madison, WI, USA). Oligonucleotides were purchased from BioTeZ Berlin-Buch GmbH (Berlin, Germany). Epothilone D was purchased via Biorbyt Ltd. (Cambridge, United Kingdom). Isopropyl ß-D-1-thiogalactopyranoside (IPTG) and 5-aminolevulinic acid were purchased from Carbolution chemicals (Saarbruecken, Germany). Bacterial media were purchased from Becton Dickinson (Heidelberg, Germany). All other chemicals were obtained from standard sources in the highest purity available.

### Plasmids and strains

The gene encoding EpoK was amplified by polymerase chain reaction (PCR) using the primers EpoK_Bamfor (CAGTGGATCCATATGACACAGGAGCAAGCGAATCAG) and EpoKhis_hindrev (ATGAAGCTTAGTGATGGTGATGGTGATGT-CCAGCTTTGGAGGGCTTCAAG) as well as Phusion^TM^ High Fidelity DNA polymerase and genomic DNA of *S. cellulosum* So ce90 as template. The PCR primers introduced a sequence encoding a hexahistidine-tag in front of the stop codon as well as the restriction sites used for cloning into pCWori+. The FNR cDNA fragment coding for the full-length mature protein of *Chlamydomonas reinhardtii* was inserted into the pET-3d plasmid between the restriction sites NcoI and BamHI giving rise to the construct pET-FNR. The construct was designed in such a way that the N- and C-termini of the recombinant protein were MASLRKPS and NQWHVEVY, respectively. The *E. coli* strain BL21 (DE3) for the heterologous expression of the P450s was purchased from Agilent Technologies (Santa Clara, CA, USA).

### Bioinformatics

Pairwise protein sequence alignments were performed using the Needlemann-Wunsch algorithm (EMBL-EBI: Needle (EMBOSS)). Protein sequences were taken from NCBI protein database (UniProtKB).

### Heterologous expression and purification of P450s

*E. coli* BL21 (DE3) cells were transformed with the pCWori_EpoK plasmid encoding the C-terminal hexahistidine-tagged EpoK from *S. cellulosum* So ce90. An overnight culture was prepared from a single colony and was grown at 37 °C in lysogeny broth (LB) medium (10 g tryptone, 5 g yeast extract and 10 g NaCl per liter H_2_O) containing 100 *μ*g ml^−1^ ampicillin. For the heterologous expression of EpoK, a main culture of 500 ml terrific broth (TB) medium (24 g yeast extract, 12 g peptone, 4 ml glycerol, 2.31 g K_2_HPO_4_ and 12.54 g KH_2_PO_4_ per liter H_2_O) containing 100 *μ*g/ml ampicillin was inoculated with 5 ml of the overnight culture and grown at 37 °C. At an optical density (600 nm) of 0.9, 1 mM IPTG and 0.5 mM 5-aminolevulinic acid were added. After 24 h of expression at 25 °C, cells were harvested by centrifugation at 4000 × g for 20 min. Cell pellets were stored at −20 °C until purification. For protein purification, a cell pellet was thawed on ice and resuspended in lysis buffer (50 mM Tris-Cl (pH 7.5) buffer with 10% glycerol, 0.5 M sodium chloride, 5 mM ß-mercaptoethanol, 5 mM imidazole and 1 mM phenylmethylsulfonyl fluoride). Cells were disrupted by sonication and the separation of the cytosolic fraction was ensured by centrifugation at 30,000 rpm for 30 min. The supernatant was loaded on a 5 PRIME* PerfectPro* Ni-NTA Agarose column (Fisher scientific, Schwerte, Germany), washed with lysis buffer (twice the volume of packed column) and eluted with a linear gradient from 5 mM to 200 mM imidazole. Fractions of 2 ml were collected and analyzed via UV-Vis spectroscopy (UV- 2101PC, SHIMADZU, Japan). Fractions showing an absorption value (A_420 nm_/A_276 nm_) higher than 1.1 were pooled, washed with storage buffer (50 mM Tris-Cl (pH 7.5) buffer with 10% glycerol, 0.5 mM dithiothreitol and 1 mM EDTA) and concentrated using a Centricon ultrafiltration unit with 30-kDa cut-off (Millipore). The concentration of EpoK was determined by recording the oxidized spectra using *ε* (420 nm–490 nm) = 110 mM^−1^ cm^−1^ 60.

The P450s CYP109C1, CYP109C2, CYP109D1, CYP260A1, CYP260B1, CYP264A1, CYP264B1, CYP265A1, CYP266A1, CYP267A1, and CYP267B1 were expressed and purified as described previously^17^,^19^,^22^,^23^,^54^.

### Heterologous expression and purification of redox partners

The electron transfer partners Adx_4-108_ and AdR from *Bos taurus* were expressed and purified as noted elsewhere^61^,^62^. The ferredoxin Etp1^fd^ and the reductase Arh1 from *S. pombe* were expressed and purified as described^28^,^29^. The ferredoxins Fdx2 and Fdx8 as well as the reductase FdR_B from *S. cellulosum* So ce56 were expressed and purified analogous to previous studies in our laboratory^31^.

*E. coli* BL21(DE3) was transformed with pET3d-FNR (ampicillin resistance) and pSBET (kanamycin resistance), the latter one encoding tRNAs for rare arginine codons^63^ and colonies with double resistance were selected. SynFdx expression was performed from an ampicillin-resistant pCK5-Fdx plasmid in *E. coli* DH5*α*^64^. In both cases the antibiotic resistant strains were grown at 37 °C in 2.4 l LB medium supplemented with the required antibiotics (ampicillin at 100 *μ*g ml^−1^ or kanamycin at 50 *μ*g ml^−1^) and in the case of SynFdx with additional 50 *μ*M FeSO_4_. Protein expression was induced at exponential phase by adding 100 *μ*M IPTG for 4 h at 37 °C. The cultures were then centrifuged for 15 min at 4400 × g. The pellets were resuspended in 30 ml of 30 mM Tris–HCl (pH 8.0), 200 mM NaCl (Tris-NaCl buffer).

Cell lysis was performed by sonication (3 × 1 min with intervals of 1 min) and the soluble and insoluble fractions were separated by centrifugation for 30 min at 35,000 × g. The soluble part was then fractionated in two steps first up to 40% of the saturation in ammonium sulfate, then up to 80% for FNR or to 90% for SynFdx. After centrifugation (20 min, 20,000 × g), the recombinant proteins in the pellets from the 40 to 80/90% ammonium sulfate fractions were purified first by size exclusion chromatography after loading onto an ACA44 (5 × 75 cm) column equilibrated in Tris-NaCl buffer. The fractions containing the highest absorption values (SynFdx: A_420 nm_/A_275 nm_; FNR: A_455 nm_/A_275 nm_) were pooled, dialyzed by ultrafiltration to remove NaCl and loaded onto a DEAE (diethylaminoethyl) cellulose column (Sigma-Aldrich, Hannover, Germany) equilibrated in a 30 mM Tris–HCl (pH 8.0) buffer. The proteins were then eluted using a 0 to 0.4 M NaCl gradient. The fractions of interest were pooled, concentrated by ultrafiltration under nitrogen pressure (Amicon, YM10 membrane) and stored in the same buffer at −20 °C at concentrations higher than 5 mg/ml. Purified *Synechocystis* ferredoxin had ratios A_420 nm_/A_275 nm_ higher than 0.5. *Chlamydomonas reinhardtii* FNR preparations had ratios A_275 nm_/A_455 nm_ of about 7. Molecular extinction coefficients of the ferredoxins and ferredoxin reductases expressed and purified during this study are summarized in supplemental Table S0.

### *In vitro* conversions

To measure the *in vitro* conversion of epothilone D to B, a reconstituted *in vitro* system analogous to Julien *et al*. and Tang *et al*. was chosen^12^,^21^. EpoK (1.5 *μ*M), the regeneration system consisting of glucose-6-phosphate (3.3 mM) and glucose-6-phosphate dehydrogenase (0.5 U), and epothilone D (100 *μ*M) were used for each sample. The ratios of EpoK to the tested ferredoxins and reductases were selected corresponding to previous studies. *In vitro* conversions with *bovine* electron transfer partners were done using a ratio EpoK:Adx_4-108_:AdR of 1:20:3^22^ and the ratio of the redox partners from *S. cellulosum* So ce56 were chosen as 1:60:3 (both EpoK:Fdx2:FdR_B and EpoK:Fdx8:FdR_B) corresponding to published values^31^. The ratio for the electron transfer system from *S. pombe* was set as 1:8:0.8 (EpoK:Etp1^fd^:Arh1) as described by Ewen *et al*.^29^. Ferredoxin from *Synechocystis* (SynFdx) was tested with different heterologous reductases (ratio EpoK:SynFdx:reductase 1:10:1 and 1:20:3). The total volume of the reaction was 200 *μ*l. The reaction was started by the addition of NADPH (1 mM). After 1 h at 30 °C the reaction was quenched by adding ethyl acetate (400 *μ*l). The aqueous phase was extracted twice with ethyl acetate (2 × 400 *μ*l), unified and evaporated to dryness. All experiments were done twice and a negative control without P450 was implemented to verify the P450-dependent reaction.

*In vitro* conversions with P450s from *S. cellulosum* So ce56 were done as described before with potassium phosphate buffer (50 mM, pH 7.4, 1% glycerin) as reaction buffer.

Kinetic studies of epothilone D epoxidation by EpoK/SynFdx/FNR system were performed analogous to the reconstituted *in vitro* system described above. EpoK concentration was set as 0.25 *μ*M with an EpoK:SynFdx:FNR ratio of 1:20:3. The reaction was stopped after 90 s at 30 °C by freezing the samples in liquid nitrogen.

### Analysis of the *in vitro* conversions via HPLC

The HPLC analysis was performed on a Jasco (Gross-Umstadt, Germany) HPLC system 2000. The samples were dissolved in 100 *μ*l acetonitrile and analyzed (samples of kinetics twice) on a reversed phase column (125/4 Nucleodur 100-5 C18ec, Macherey Nagel, Düren, Germany) at a flow rate of 1 ml min^−1^ and a temperature of 40 °C with the gradient: 80% solvent A (80:20 (v/v) water-acetonitrile) for 1 min, linear gradient for 8.5 min from 20% to 90% solvent B (100% acetonitrile) and holding 90% solvent B for 4 min. The injection volume was set as 20 *μ*l (40 *μ*l for kinetic studies) and the sample was monitored at 250 nm.

### Analysis of the *in vitro* conversions via LC-MS/MS

Mass spectrometric analyses were performed using a Thermo-Fischer (Sunnyvale, CA, USA) Dionex UltiMate 3000 ultra-high performance liquid chromatography (UHPLC) system coupled to an AB Sciex (Concord, ON, Canada) QTRAP 5500 quadrupole linear ion trap (QqLIT) system. Samples were separated on a reversed-phase HPLC column (125/4 Nucleodur 100-5 C18ec, Macherey Nagel) at 40 °C using gradient elution at 0.5 mL min^−1^. The mobile phase consisted of 80:20 (v/v) water-acetonitrile (A) and 100% acetonitrile (B). The gradient was as follows: B was increased from 20 to 90% within 17 min, held there for 5 min, and then returned to 20% within 0.1 min, followed by an equilibration period at 20% B for 7.9 min. For each analysis, 10 *μ*l were injected. ESI-MS conditions were as follows: curtain gas: 55 psi, electrospray voltage: 5 kV, source heater temperature: 350 °C, ion source gas 1: 45 psi, ion source gas 2: 50 psi, collision gas: nitrogen, declustering potential: 48 V, entrance potential: 11 V. MS/MS experiments were performed using collision-induced dissociation (CID) between 15–35 V for the precursor ion *m/z* 508. The collision cell exit potential was 13 V; an isolation width of 1 u was used.

## Additional Information

**How to cite this article**: Kern, F. *et al*. Highly Efficient CYP167A1 (EpoK) dependent Epothilone B Formation and Production of 7-Ketone Epothilone D as a New Epothilone Derivative. *Sci. Rep*. **5**, 14881; doi: 10.1038/srep14881 (2015).

## Supplementary Material

Supplementary Information

## Figures and Tables

**Figure 1:**
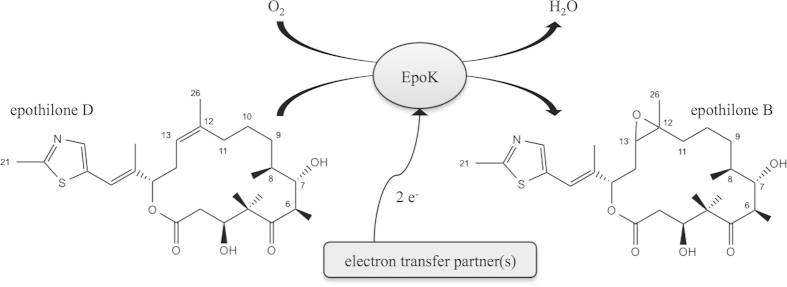
Conversion of epothilone D to B catalyzed by EpoK in *S. cellulosum* So ce90; electron transfer partners are unknown.

**Table 1:** Origins and properties of selected ferredoxins. (/: not described).

Name	Organism	Aminoacids	Molecularweight [kDa]	Redoxpotential[mV]	Fe-Sclustertype	Literature
Adx_4-108_	*Bos taurus*	104	11.8	−344	[2Fe-2S]	62,66
Etp1^fd^	*S. pombe*	127	14.1	−353	[2Fe-2S]	67
Fdx2	*S. cellulosum* Soce56	101	11.2	/	[3Fe-4S]	31
Fdx8	*S. cellulosum* Soce56	107	11.9	/	[3Fe-4S]	31
SynFdx	*Synechocystis*	96	10.3	−380	[2Fe-2S]	35,68

**Table 2:** Origins and properties of investigated reductases.

Name	Organism	Aminoacids	Molecularweight[kDa]	Literature
AdR	*Bos taurus*	492	50.3	69
Arh1	*S. pombe*	469	51.4	29
FdR_B	*S. cellulosum* So ce56	244	26.7	31
FNR	*C. reinhardtii*	320	36.5	38,70,71,72

The listed reductases contain flavin adenine dinucleotide and use both NADPH and NADH as a cofactor (Arh1 and FdR_B) with the preference for NADPH for FdR_B^31^, while AdR and FNR use NADPH only.

**Table 3:** Epothilone D conversion with selected P450s of *S. cellulosum* So ce56 (/: no conversion; *: combined yields of 26-OH epothilone D and epothilone B).

P450	Product	Retentiontime [min]	Conversion of epothilone D [%] with		
			Adx_4-108_/AdR	Fdx2/FdR_B	Fdx8/FdR_B
EpoK	Epothilone B	6.3	6.8	24.7	74.4
CYP265A1	14 OH-epothilone D	5.5	3.2	5.5	6.0
CYP266A1	14 OH-epothilone D	5.5	/	6.9	2.6
CYP267B1	21-OH epothilone D	5.1	3.5	3.2	5.5
	14-OH epothilone D	5.5	3.5	3.4	4.8
	26-OH epothilone D/epothilone B	6.3	11.6*	4.0*	11.7*
	Not furthercharacterized product	9.5	3.9	2.0	4.8
	7-ketone epothilone D	10.9	6.6	9.3	8.7
