# Supplementary material for: Highly Efficient CYP167A1 (EpoK) dependent Epothilone B Formation and Production of 7-Ketone Epothilone D as a New Epothilone Derivative
Source: Sci Rep. 2015 Oct 8;5:14881. doi: 10.1038/srep14881 (PMC4597204; doi:10.1038/srep14881)
Supplement: Supplementary Information [file srep14881-s1.pdf]

SUPPORTING INFORMATION

Highly efficient CYP167A1 (EpoK) dependent epothilone B formation and production of 7-ketone epothilone D as a new epothilone derivative

Fredy Kern[1], Tobias K. F. Dier[2], Yogan Khatri[1], Kerstin M. Ewen[1], Jean-Pierre Jacquot[3], Dietrich A. Volmer[2] and Rita Bernhardt[1]

[1]From the Department of Biochemistry, Saarland University, 66123 Saarbrücken, Germany

[2]Institute of Bioanalytical Chemistry, Saarland University, 66123 Saarbrücken, Germany

[3]Unité Mixte de Recherches, 1136 Interaction arbres microorganismes INRA, Nancy University, 54506 Vandoeuvre-lès-Nancy cedex, France

To whom correspondence should be addressed: Rita Bernhardt, Department of Biochemistry, Saarland University, 66123 Saarbrücken, Germany, Tel.: +49 (0)681 302 4241; Fax: +49 (0)681 302 4739; E-mail: ritabern@mx.uni-saarland.de

Molecular extinction coefficients of ferredoxins and ferredoxin reductases - The summarized extinction coefficients of ferredoxins and ferredoxin reductases expressed and purified in this study are listed in Table S0.

Table S0. Overview: molecular extinction coefficients of ferredoxins and ferredoxin reductases. (Values were taken from literature as referred in Table 1 and Table 2; results section)

**Ferredoxin**	**Organism**	**Extinction coefficient [mM^{-1} cm^{-1}]**	**Reductase**	**Organism**	**Extinction coefficient [mM^{-1} cm^{-1}]**
Adx_{4-108}	*Bos taurus*	ε_{414nm}: 9.8	AdR	*Bos taurus*	ε_{450nm}: 11.3
$Etp1^{fd}$	*S. pombe*	ε_{414nm}: 9.8	Arh1	*S. pombe*	ε_{450nm}: 11.3
Fdx2<br>Fdx8	*S. cellulosum So ce56*	ε_{390nm}: 6.181<br>ε_{400nm}: 9.7	FdR_B	*S. cellulosum* So ce56	ε_{457nm}: 8.73
SynFdx	*Synechocystis*	ε_{422nm}: 9.7	FNR	*C. reinhardtii*	ε_{450nm}: 11.3

Structure of products - During this study, 4 new P450-derived epothilone D derivatives were found and characterized via LC-MS/MS. An overview of the chemical structures is presented in Figure S1.

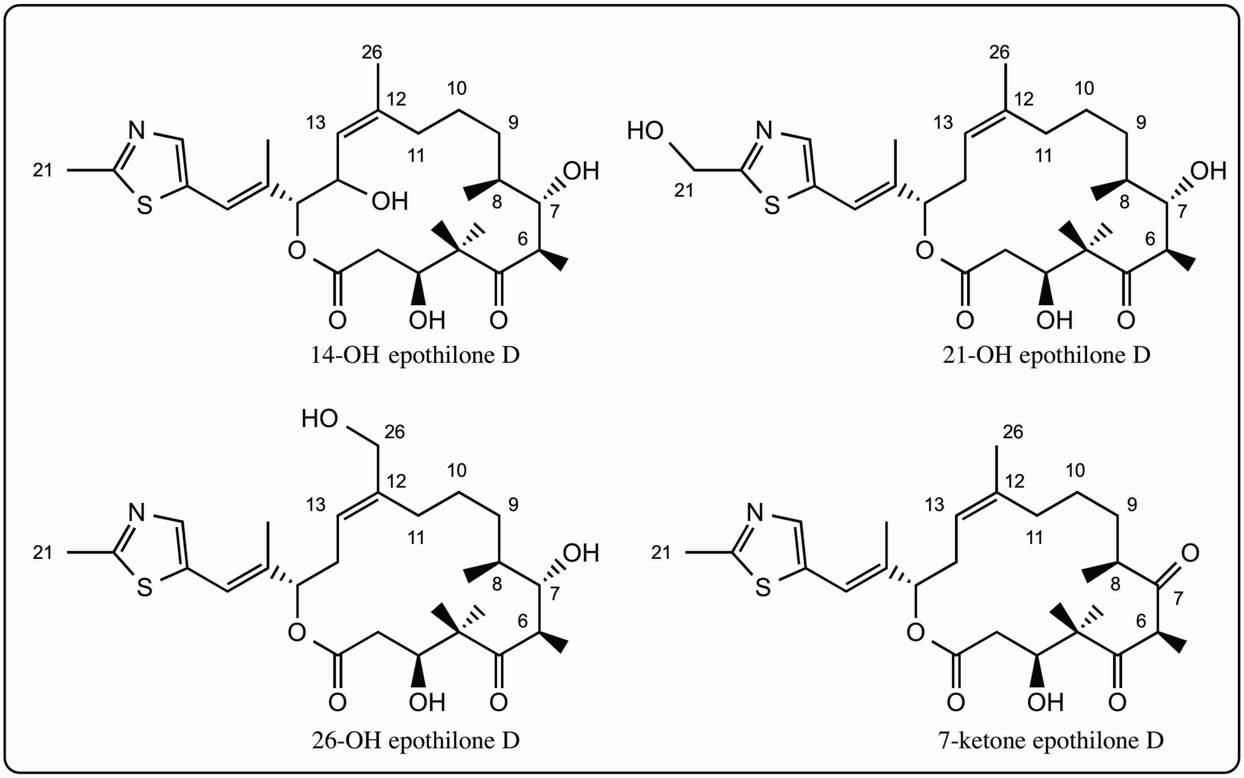

Figure S1. Overview of major epothilone D products formed by myxobacterial P450s from *S. cellulosum* So ce56 (CYP265A1 and CYP266A1: 14-OH epothilone D; CYP267B1: 14-OH, 21-OH, 26-OH and 7-ketone epothilone D).

LC-MS/MS data - The chromatograms of LC-MS/MS experiments are presented in Figure S2. Products A and B are already labeled as 21-OH epothilone D as well as product C (14-OH epothilone D), product D (26-OH epothilone D) and product E (7-ketone epothilone D).

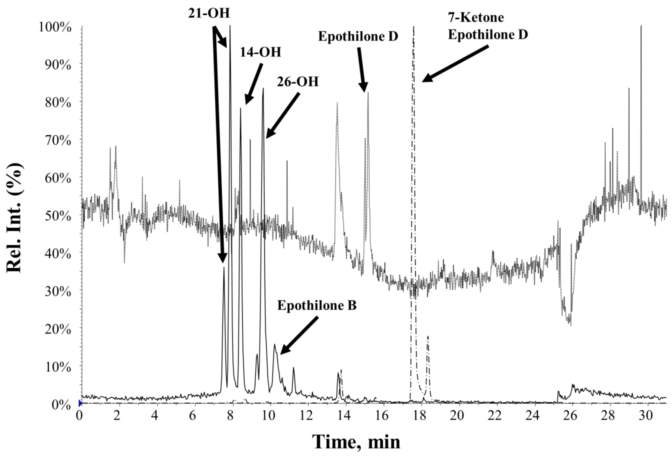

Figure S2. LC-MS/MS chromatograms of epothilone D conversion.

The MS/MS spectrum of the new epothilone derivative 7-ketone epothilone D is presented in Figure S3.

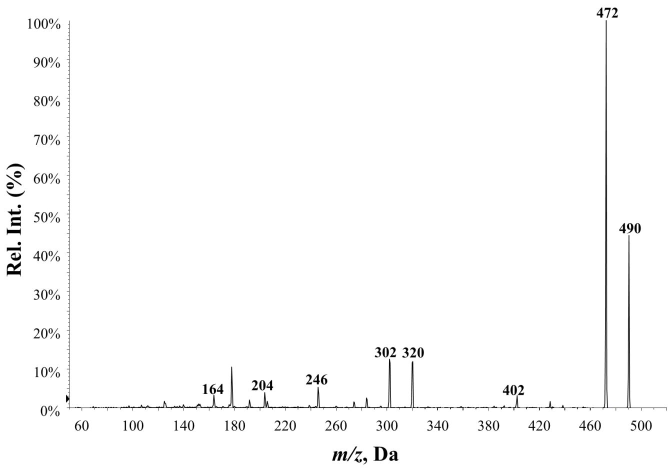

Figure S3. MS/MS spectrum of 7-ketone epothilone D.

An overview of the chemical structures assigned to the fragments observed in the LC-MS/MS spectra of the identified products is listed in Table S1. For further information on 7-ketone epothilone D, see Figure 5 in the discussion section.

Table S1. Overview of conversion products tentatively identified by LC-MS/MS (a: taken from [37]; *: fragments observed in 14-OH, 21-OH, 26-OH and 7-ketone epothilone D MS/MS spectra; b: see Figure 5 in the manuscript for fragment structures).

Name (*m/z* of $[M+H]^+$)	Chemical structure	MS^2/CID product ions (*m/z*)
Epothilone B (508)	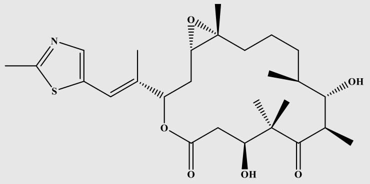	508-H_2O (490)[a*]; 490-H_2O (472)[*]; 508-$C_3H_4O_3$ (420)[a*]; 420-H_2O (402)[*]; 508-$C_7H_{12}O_5$ (332)[a*]; 508-$C_8H_{14}O_4$ (320)[a*]; 320-H_2O (302)[a*]; 166[a*]
14-OH epothilone D (508)	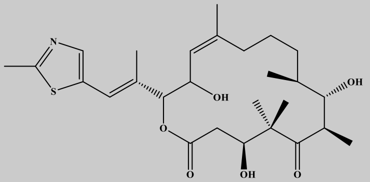	206 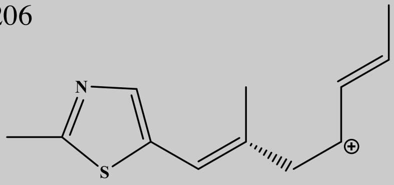
21-OH epothilone D (508)	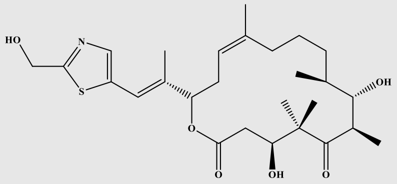	168 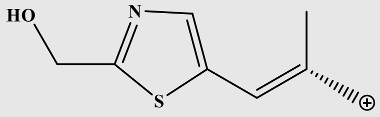
26-OH epothilone D (508)	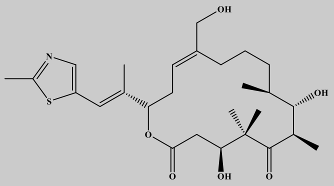	220 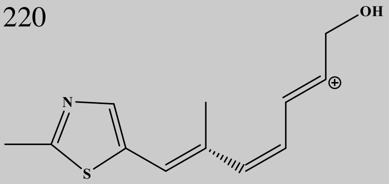
7-Ketone epothilone D (490)	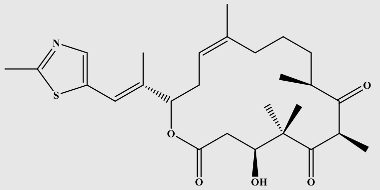	490-H_2O (472)[b]; 490-CO_2,C_2H_4O; (402)[b]; 320[b]; 246[b]; 204[b]; 164[b]

Bioinformatic analysis of the putative Fdx2, Fdx8 and FdR_B-like ferredoxins and ferredoxin reductase of S. cellulosum So0157-2 - Among UniProtKB Bacteria database, the Proteome of *Sorangium cellulosum* So0157-2 (GenBank: CP003969.1, length = 14782125) was found producing significant alignments with protein sequences of Fdx2, Fdx8 and FdR_B from *S. cellulosum* So ce56 (NCBI BLAST+). Pairwise protein sequence alignments were performed using the Needlemann-Wunsch algorithm (EMBL-EBI: Needle (EMBOSS).

Table S2. Summarized alignment results of Fdx2, Fdx8 and FdR_B from *S. cellulosum* So ce56 with *S. cellulosum* So0157-2.

**Protein (So ce56)**	**Gene name in So0157-2**	**Identity**	**Similarity**	**Gaps**
Fdx2	SCE1572_46000 [4Fe-4S] ferredoxin	94/101 (93.1%)	98/101 (97.0%)	0/101 (0.0%)
Fdx8	SCE1572_33470 Hypothetical protein	86/110 (78.2%)	89/110 (80.9%)	11/110 (10.0%)
FdR_B	SCE1572_31190 Hypothetical protein	227/245 (92.7%)	235/245 (95.9%)	1/245 (0.4%)
